# Arecoline Induces ROS Accumulation, Transcription of Proinflammatory Factors, and Expression of KRT6 in Oral Epithelial Cells

**DOI:** 10.3390/biomedicines12020412

**Published:** 2024-02-09

**Authors:** Tong-Hong Wang, Yen-Wen Shen, Hsin-Ying Chen, Chih-Chieh Chen, Nan-Chin Lin, Yin-Hwa Shih, Shih-Min Hsia, Kuo-Chou Chiu, Tzong-Ming Shieh

**Affiliations:** 1 Biobank, Chang Gung Memorial Hospital, Taoyuan 33305, Taiwan; 2 School of Dentistry, China Medical University, Taichung 404328, Taiwan; 3 Department of Sports Medicine, China Medical University, Taichung 404328, Taiwan; 4 Department of Oral and Maxillofacial Surgery, Show Chwan Memorial Hospital, Changhua 505029, Taiwan; 5 Department of Oral and Maxillofacial Surgery, Changhua Christian Hospital, Changhua 500011, Taiwan; 6 Department of Healthcare Administration, Asia University, Taichung 41354, Taiwan; 7 School of Nutrition and Health Sciences, College of Nutrition, Taipei Medical University, Taipei 110301, Taiwan; 8 Division of General Dentistry, Taichung Armed Forces General Hospital, Taichung 411228, Taiwan; 9 School of Dentistry, National Defense Medical Center, Taipei 114201, Taiwan

**Keywords:** arecoline, interleukin-6, keratin 6, reactive oxygen species, tumor necrosis factor-α

## Abstract

Areca nut is a major contributor to the high prevalence of oral cancer in Asia. The precise mechanisms by which areca nut stimulates mucosal cells and contributes to the progression of oral cancer urgently require clarification. The current study aimed to assess the effects of arecoline on the normal human gingival epithelium cell line S-G. Cell viability, levels of reactive oxygen species (ROS), protein expression, cellular morphology, and gene expression were evaluated using the MTT test, flow cytometry, Western blot analysis, optical or confocal microscopy, and RT-qPCR. Keratin (KRT6) analysis involved matched normal and cancer tissues from clinical head and neck specimens. The results demonstrated that 12.5 µg/mL of arecoline induced ROS production, tumor necrosis factor-α (TNF-α), and interleukin-6 (IL-6) mRNA expression in S-G cells. This activation of the MAPK/ERK pathway increased KRT6 expression while limiting cell migration. In head and neck cancer tissues, *KRT6B* gene expression exceeded that of normal tissues. This study confirms that arecoline induces ROS accumulation in normal cells, leading to the secretion of proinflammatory factors and KRT6 expression. This impedes oral mucosal healing, thereby promoting the progression of oral cancer.

## 1. Introduction

Chewing areca nut is a primary risk factor for oral cancer in Melanesia, South–Central Asia, and Southeastern Asia [1,2,3,4,5]. Derived from the seed of the betel palm (Areca catechu), areca nut is consumed by approximately 600 million people globally. The composition of betel quid (BQ) can vary among different countries. A common practice is wrapping betel nuts in leaves from the betel pepper plant (Piper betel) and combining them with tobacco, slaked lime (calcium hydroxide), and occasionally other spices [6]. Individuals who regularly chew areca nut commonly experience the formation of oral mucosal ulcers and these wounds often exhibit a slow healing process. The delayed healing of oral mucosal wounds and the recurrence of inflammation are observed phenomena in the progression of oral carcinogenesis induced by areca nut consumption. The process of wound healing involves a complex interplay among various cell types, cytokines, mediators, and the vascular system [7]. Wound healing involves three phases: the inflammatory phase, proliferative phase, and maturation phase. Inflammation induced by exogenous environmental stimuli can promote tumor development and progression [8,9,10]. However, the extract from betel quid demonstrates anti-inflammatory effects and enhances wound re-epithelialization during the mucosal wound healing process [11]. Arecoline is a major alkaloid component of areca nuts. Different doses of arecoline induce various cellular phenotypes, including cell proliferation, DNA damage, DNA repair ability, cell cycle arrest, apoptosis, and epithelial–mesenchymal transition (EMT). [12,13,14,15]. Hence, cells may exhibit diverse responses depending on the concentration and duration of exposure to areca nut.

Cytokines are multifunctional molecules that act as mediators, playing a crucial role in initiating or influencing various biological processes, such as inflammation, sepsis, and wound healing [16]. The induction of reactive oxygen species (ROS) production is triggered by tumor necrosis factor-α (TNF-α). Following this, ROS activates NF-κB signaling through IκB degradation, leading to NF-κB p65 nuclear translocation. This process increases mRNA expression of interleukin-6 (IL-6) and TNF-α itself. [17]. Basal cell keratinocyte proliferation is mediated by the autocrine and paracrine TNF-α [18]. IL-6 has been demonstrated to play a crucial role in epithelialization and influence granulation tissue formation [19]. Betel nut users exhibit elevated ROS levels in their saliva. Arecoline amplifies ROS and cytokine expression, contributing to cancer development and its progression to a metastatic stage. [20]. The concentrations of 0.2 mM and 0.4–1.2 mM arecoline exhibited mild stimulatory and marked suppressive effects, respectively, on IL-6 production by gingival keratinocytes (GK). Additionally, the concentrations of 0.1–1.2 mM arecoline showed minimal up-regulation of TNF-α production by GK. [21].

In response to injury in the interfollicular epidermis, there is a substantial up-regulation of keratin 6 (KRT6), which is a type II intermediate filament (IF) protein. The lack of KRT6 expression can impact wound healing [22]. Paradoxically, the absence of KRT6 isoforms leads to accelerated directional cell migration [23]. In specimens from patients with oral squamous cell carcinoma (OSCC), the expression of KRT1, KRT5/6, KRT8/18, KRT10, KRT14, and KRT19 was observed. Among these, the expression of KRT5/6 exhibited the most significant variation in OSCC tissues [24]. KRT6 and KRT16 are proposed as potential biomarkers for diagnosing head and neck squamous cell carcinoma (HNSCC) [25]. Both KRT6 and KRT1 belong to the type II keratins. The induction of KRT1 expression may be associated with intracellular oxidative stress [26]. Currently, it is unclear whether areca nut regulates KRT6 gene expression, impacting wound healing and cancer progression in normal oral cells. Therefore, this study aims to investigate the impact of arecoline-induced reactive oxygen species (ROS) on the expression of proinflammatory cytokine genes and KRT6 in the normal gingival epithelial cell line S-G in vitro. Additionally, we will verify the gene expression of KRT6 in oral cancer patients.

## 2. Materials and Methods

### 2.1. Cell Culture

Smulow–Glickman cells (S-G cells, an immortalized human gingival epithelial cell line) were cultured in DMEM containing 10% fetal bovine serum and 1% penicillin/streptomycin [27]. The S-G cell line was kindly gifted by Professor Hsi-Feng Tu, (National Yang Ming Chiao Tung University, Taipei, Taiwan). When cell confluency reached 90%, cells were treated with various concentrations of arecoline for different durations. To investigate whether the expression levels of KRT6 protein are regulated by the JNK or MEK signaling pathway, cells were pretreated with or without inhibitors for JNK (SB600125, 10 μM) or MAPK/ERK (PD98059, 10 μM) for 2 h before adding arecoline to the cells.

### 2.2. Subjects

This study included paired samples from 49 cases of HNSCC, diagnosed between 10 August 2007 and 16 September 2019, obtained from Changhua Christian Hospital Tissue Bank. The HNSCC encompassed 20 cases of oral squamous cell carcinoma (OSCC), 12 cases of oropharyngeal squamous cell carcinoma (OPSCC), 15 cases of laryngeal squamous cell carcinoma (LSCC), and 2 cases of other types. The Institutional Ethics Committee approved the study on 29 March 2022 (Approval No: 200501). Tissue samples were stored in liquid nitrogen until further analysis. A minimum of 70% tumor cells in selected frozen sections of HNSCC samples was required for the investigation.

### 2.3. Cell Viability

S-G cells were seeded at a density of 5 × 10^3^ cells/well in 96-well culture plates one day before conducting cell viability assays. Cells were treated with or without arecoline (12.5 and 25 μg/mL) for 24 h or with 12.5 μg/mL for 1 to 5 days. At the end of each time point, 3-(4,5-dimethylthiazol-2-yl)-2,5-diphenyltetrazolium bromide (MTT) assays were performed and the optical density values were detected at 590 nm using an ELISA reader (VarioSkan Lux, thermoscientific) according to the previously reported protocol [28].

### 2.4. Detection of Intracellular ROS

S-G cells were seeded at a density of 5 × 10^5^ cells/well in 6-well culture plates and treated with arecoline (12.5 μg/mL) for 2 and 4 h. Subsequently, the cells were incubated in culture medium containing 20 µM of 5-(and 6)-Carboxy-2′,7′-dichlorodihydrofluorescein diacetate (carboxy-H2DCFDA) at 37 °C for 60 min in the dark. The intracellular ROS levels were detected using a flow cytometer (Thermo Fisher Scientific, Waltham, MA., USA) or a fluorescence microscope (Eclipse TS100, Nikon, Kōnan, Minato, Tokyo, Japan) [13].

### 2.5. Western Blot Analysis

Cell lysis was performed with RIPA buffer containing a protease and phosphatase inhibitor cocktail (Roche, Basel, Switzerland). Equal amounts of proteins were separated by SDS-PAGE before transferring to PVDF membranes. After blocking with 5% non-fat milk for 1 h, the membranes were incubated with primary antibodies catalase, CDK1, cyclin B1, E-cadherin, GAPDH, GPx4, keratin 6, Nrf-2, p21, p-ERKthr202/tyr204, p-IKBαser32/36, p-JNKthr183/tyr185, SOD1, and vimentin (refer to Supplementary Table S1) at 4 °C overnight. After washing with PBS, the membranes were hybridized with anti-rabbit or mouse horseradish peroxidase (HRP)-conjugated IgG secondary antibodies (Abcam, Cambridge, UK) for 1 h at 25 °C. Chemiluminescence signals, generated by adding ECL reagents (Thermo Fisher Scientific, Waltham, MA., USA), were detected using the eBlot Touch Imager (eBlot Photoelectric Technology, China). Protein bands in the blots were quantified using ImageJ V 1.8.0 software (National Institutes of Health, Bethesda, MD, USA).

### 2.6. Reverse Transcription and Quantitative Real-Time PCR Analysis (RT-qPCR)

Total RNA was extracted using TRIzol™ Reagent (Invitrogen, Waltham, MA, USA), and mRNA levels were quantified at absorbances of 260 and 280 nm using a GeneQuant 1300 Spectrophotometer (Biochrom US, Holliston, MA, USA). The cDNA was reverse transcribed from 1 µg of total RNA using M-MLV reverse transcriptase (Invitrogen, Waltham, MA., USA), followed by mixing with specific primers and 2X Universal SYBR Green Fast qPCR Mix (ABclonal, Düsseldorf, Germany). The primer sequences are detailed in Supplementary Table S2. mRNA levels were analyzed using the Applied Biosystems StepOnePlus™ Real-Time PCR System (Thermo Fisher Scientific, Waltham, MA, USA). All reactions were performed in triplicate, and relative gene expressions were quantified by normalizing to that of *GAPDH* and calculated using the 2^−∆∆Ct^ method [29].

### 2.7. Immunocytochemistry Using Confocal Microscopy

S-G cells were pretreated with or without SP600125 (10 μM) and PD98059 (10 μM) for 2 h, followed by the addition of arecoline (12.5 μg/mL) and incubation for another 24 h. Cells were fixed in 10% formaldehyde for 15 min, then treated with 0.1% Triton X-100 for 10 min. After washing three times with cold PBS, cells were blocked in 1% bovine serum albumin for 1 h at room temperature. Subsequently, cells were incubated with a primary antibody recognizing KRT6 at 4 °C overnight. Following this, cells were incubated with m-IgGk BP-CFL 488 (sc-516176, Santa Cruz, Dallas, TX., USA), and Hoechst 33258 (H3569, Thermo Fisher Scientific, Waltham, MA., USA) was used to stain the nuclei for 1 h at room temperature. Fluorescence images of KRT6 expression were acquired using a confocal microscope (Leica SP8X Confocal Spectral Microscope, Wetzlar, Germany) [30].

### 2.8. Wound Healing Assay

To create wounds, 5 × 10^5^ cells were seeded in 6-well plates one day before sterile pipette tips were used to draw a line in the middle of each well. Cells were then treated with 12.5 μg/mL of arecoline for 48 h. Each well was washed twice with PBS, and wound areas were observed and photographed using an inverted microscope. The widths of the wounds were quantified using ImageJ software (National Institutes of Health, Bethesda, MD, USA) [30].

### 2.9. Statistical Analysis

All experiments were conducted a minimum of three times. The experimental results were analyzed using Student’s *t*-test (SPSS 12.0 software, IBM, Armonk, NY, USA), and a *p*-value less than 0.05 was deemed statistically significant.

## 3. Results

### 3.1. Arecoline Treatment Immediately Induced Up-Regulation of ROS and Proinflammatory Cytokines in S-G Cells

**Figure 1:**
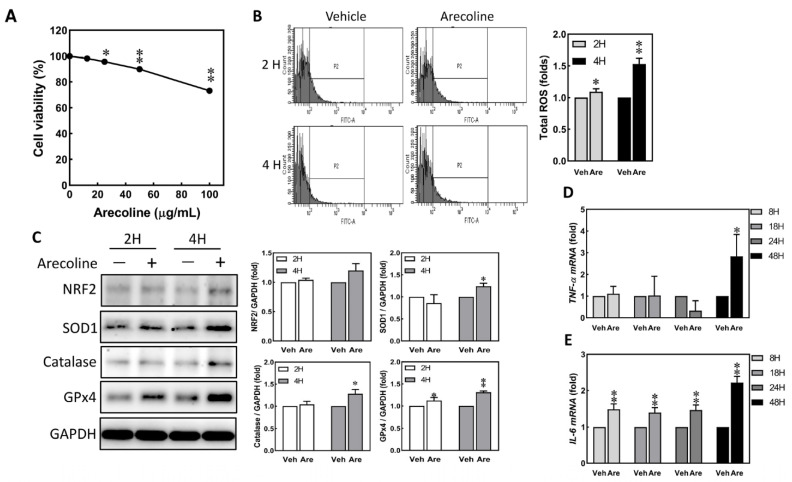
Arecoline treatment immediately induced up-regulation of ROS and proinflammatory cytokines in S-G cells. The S-G cells were treated with vehicle (DMSO) and various doses of arecoline for 2 to 24 h. (**A**) Cell viability of S-G cells was assessed after treatment with 0, 12.5, 25, 50, and 100 μg/mL of arecoline for 24 h. (**B**) ROS changes in S-G cells were evaluated after exposure to 0 and 12.5 μg/mL of arecoline for 2 and 4 h. Left, flow cytometry; right, quantitative results. (**C**) Expression of antioxidant proteins in S-G cells after treatment with 0 and 12.5 μg/mL of arecoline for 2 and 4 h. GAPDH was used as the loading control. Left, Western blotting; right, quantitative results. The mRNA expression of *TNF-α* (**D**) and *IL-6* (**E**) was evaluated after exposure to 0 and 12.5 μg/mL of arecoline for 8, 18, 24, and 48 h. Columns, mean of more than triplicate analysis; bars, SE. * *p* < 0.05 and ** *p* < 0.01 indicate the significance levels of the arecoline treatment groups compared to the control group.

S-G cells were treated with vehicle (DMSO) and 12.5 to 100 μg/mL of arecoline for 24 h; arecoline doses below 12.5 μg/mL showed no significant difference compared to the vehicle (Figure 1A). Exposure to a low dose of 12.5 μg/mL of arecoline for 2 and 4 h induced the accumulation of intracellular reactive oxygen species (ROS) in S-G cells (Figure 1B). Additionally, antioxidative stress proteins, including NRF2, SOD1, catalase, and GPx4, were significantly up-regulated after treating with 12.5 μg/mL of arecoline for 2 or 4 h (Figure 1C). To investigate whether the increase in ROS originates from TNF-α and promotes downstream IL-6 expression, RT-qPCR analysis will be conducted to assess the transcriptional activity of *TNF-α* and *IL-6* genes during 8–48 h. While arecoline treatment up-regulated the expression levels of *IL-6* in all four time points, the levels of *TNF-α* did not increase until S-G cells were treated with arecoline for 48 h (Figure 1D,E). Therefore, the observed accumulation of ROS induced by arecoline at 4 h may involve mechanisms other than TNF-α induction of ROS.

### 3.2. Arecoline Treatment Induced the Expression of KRT6 in S-G Cells

**Figure 2:**
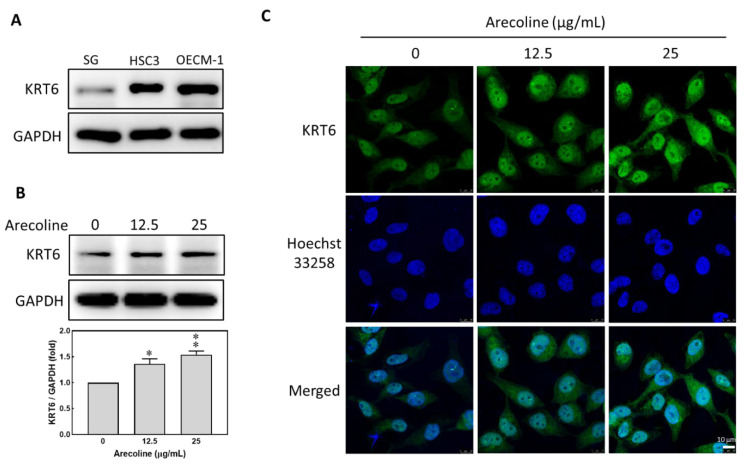
Arecoline treatment induced the expression of KRT6 in S-G cells. (**A**) The protein expression levels of KRT6 in S-G, HSC3, and OECM-1 cells. GAPDH was used as the loading control. (**B**) Expression of KRT6 proteins in S-G cells after treatment with 0, 12.5, and 25 μg/mL of arecoline for 24 h. Above, Western blotting; below, quantitative results. (**C**) Fluorescent images of S-G cells treated with 0, 12.5, and 25 μg/mL of arecoline were analyzed by a confocal microscope. Green represents KRT6; blue represents Hoechst 33258. Lower right corner, scale bar, 10 µm. Columns, mean of more than triplicate analysis; bars, SE. * *p* < 0.05 and ** *p* < 0.01 denote the significance levels of arecoline treatment groups compared to the control group.

To investigate whether KRT6 is expressed in noncancerous and cancerous cells, the protein levels of KRT6 in immortalized human gingival oral epithelial S-G cells and oral squamous cell carcinoma cell lines HSC3 and OECM-1 cells were analyzed using Western blot analysis. The protein levels of KRT6 in the oral cancer cells were higher than those in S-G cells (Figure 2A). The effect of arecoline on inducing KRT6 expression in S-G cells was evaluated. Arecoline concentrations of 12.5 to 25 μg/mL resulted in less than 5% cell death. However, treatment with arecoline (12.5 μg/mL) for 24 h up-regulated the protein expression levels of KRT6 in S-G cells (Figure 2B). Observation under confocal microscopy revealed that the KRT6 protein expression was localized in the nucleus and cytoplasm, and the KRT6 fluorescence increased in a dose-dependent manner after arecoline treatment for 24 h (Figure 2C). Therefore, a lower concentration of arecoline is able to stimulate the up-regulation of KRT6.

### 3.3. Arecoline Treatment Compromised the Abilities of Migration and Cell Proliferation in S-G Cells

**Figure 3:**
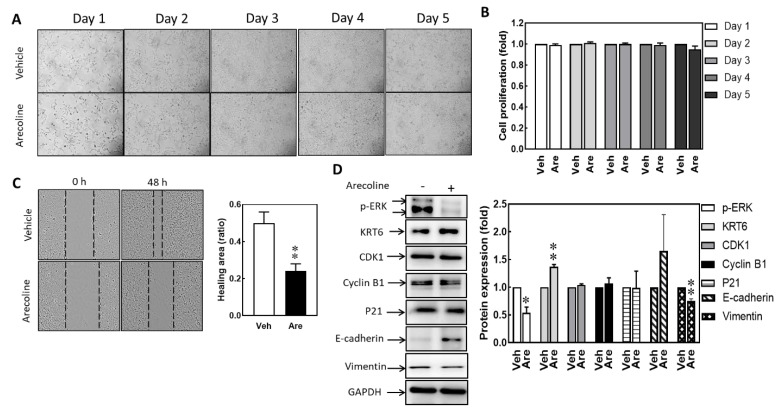
Arecoline treatment compromised the abilities of migration and cell proliferation in S-G cells. S-G cells were treated with vehicle and 12.5 μg/mL of arecoline for 1 to 5 days. (**A**) Cell morphology. (**B**) Cell viability. (**C**) Wound healing assay; S-G cells were treated with vehicle and 12.5 μg/mL of arecoline for 48 h. At 0 h, the tip scraped an area without cells; at 48 h, the cell-free area showed recovery. Left: magnification is 200× and the dashed line indicates the border of the cell-free area. Right: the quantified results of wound healing. (**D**) The protein expression levels of phospho-ERK, KRT6, CDK1, cyclin B1, and P21 in S-G cells treated with vehicle or 12.5 μg/mL of arecoline for 24 h were analyzed using Western blot analysis. GAPDH was used as the loading control. Left, Western blot; right, quantitative results. Columns, mean of more than triplicate analysis; bars, SE. * *p* < 0.05 and ** *p* < 0.01 are the significance levels of arecoline treatment groups compared to the vehicle control group.

Stimulating S-G cells with 12.5 μg/mL of arecoline induces ROS production, activating IL-6 gene transcription within 8 h and TNF-α gene transcription within 48 h. The generation of ROS can lead to cell cycle arrest and DNA damage, ultimately resulting in cell death. The influence of arecoline on the proliferation and motility abilities of S-G cells was also evaluated. Treating S-G cells with 12.5 μg/mL of arecoline for 1 to 5 days showed no significant differences in cell morphology (Figure 3A). The MTT assay is employed to measure cellular metabolic activity, serving as an indicator of cell viability, proliferation, and cytotoxicity. The MTT test results indicate that, after treatment with 12.5 μg/mL of arecoline for 1–5 days, the cell proliferation ability is approximately equal to the control group (Figure 3B). Moreover, the wound healing experiment shows that the effect of arecoline inhibits healing. Therefore, the impact on cell proliferation was excluded. The cell migration activity was suppressed by arecoline treatment (Figure 3C). The Western blot results indicate that, when treated with 12.5 μg/mL of arecoline for 24 h, the promotion of KRT6 increase does not significantly alter cell-cycle-related proteins CDK1, cyclin B1, and P21. However, there is an enhancement in E-cadherin associated with intercellular adhesion and a decrease in vimentin associated with cell migration (Figure 3D). The changes in these protein molecules correspond to alterations in cell phenotypes.

### 3.4. Arecoline Treatment Up-Regulated KRT6 Protein Expression via ERK–NFκB Signaling Pathway

**Figure 4:**
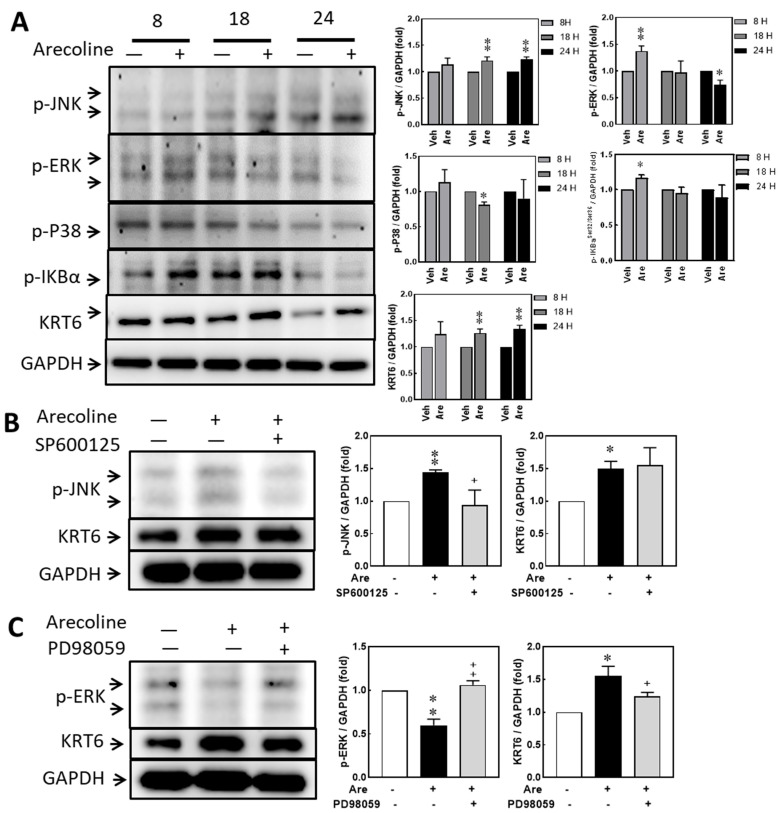
Arecoline treatment up-regulated KRT6 protein expression via ERK–NFκB signaling pathway. (**A**) S-G cells were treated with arecoline (12.5 μg/mL) for 8, 18, and 24 h. The protein expression levels of p-JNK, p-ERK, p-38, p-IKBα^ser32/36^, and KRT6 were determined by Western blot analysis. Left: Western blot; right: quantitative results. S-G cells were pretreated with or without a JNK inhibitor (SP600125, 10 μM) (**B**) or an MEK inhibitor (PD98059, 10 μM) (**C**) for 2 h before incubating with 12.5 μg/mL of arecoline for 24 h. Protein expression levels of p-JNK, p-ERK, and KRT6 were analyzed using Western blot analysis. GAPDH was used as the loading control. Right, Western blot; left, quantitative results. Columns, mean of more than triplicate analysis; bars, SE. * *p* < 0.05 and ** *p* < 0.01 indicate the significance levels of arecoline treatment groups compared to the control group. + *p* < 0.05 and ++ *p* < 0.01 indicate the significance levels compared within the arecoline-treated groups.

A previous study indicated that ROS can activate NF-κB via MAPK signaling [31]. The protein expression levels of phosphorylated JNK (p-JNK), phosphorylated ERK (p-ERK), phosphorylated P38 (p-P38), phosphorylated IκBα (p-IκBα), and KRT6 showed no significant differences between vehicle and 12.5 μg/mL arecoline treatments. Arecoline treatment induced p-JNK expression at 18 h and 24 h, induced p-ERK at 8 h, reduced p-P38 at 18 h, induced p-IκBα^ser32/36^ at 8 h, and induced KRT6 at 18 h and 24 h. The levels of p-JNK and p-ERK increased and reduced, respectively, after 24 h of arecoline stimulation (Figure 4A). Therefore, KRT6 expression was hypothesized to be correlated with the p-ERK and p-IκBα^ser32/36^-mediated signaling pathway. A JNK inhibitor (SP600125) and a MAP kinase kinase (MEK) inhibitor (PD98059) were used to confirm whether MAPK/ERK or JNK regulates KRT6 expression. The levels of arecoline-induced p-JNK and reduced p-ERK were consistent with the results in Figure 4A. However, the level of arecoline-induced KRT6 showed no significant difference in the SP600125 pretreatment group (Figure 4B) but decreased in the PD98059 pretreatment group (Figure 4C). Therefore, arecoline-induced KRT6 expression is regulated through the MAPK/ERK signaling pathway in S-G cells.

### 3.5. Inhibiting JNK and MAPK/ERK Suppresses Arecoline-Induced TNF-α Transcription but Inhibiting JNK Enhances Arecoline-Induced IL-6 Transcription in the S-G Cells

**Figure 5:**
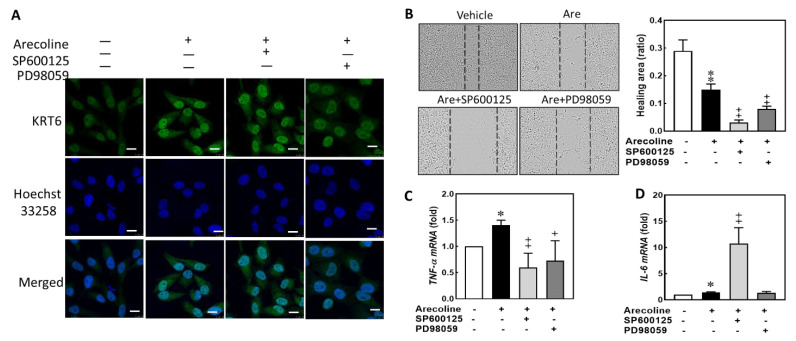
Inhibiting JNK and MAPK/ERK suppresses arecoline-induced *TNF-α* transcription but inhibiting JNK enhances arecoline-induced *IL-6* transcription in the S-G cells. S-G cells were pretreated with or without 10 μM SP600125 or 10 μM PD98059 for 2 h before incubating with 12.5 μg/mL of arecoline for 24 h. (**A**) Confocal microscope. Green, KRT6; blue, Hoechst 33258. Each white bar is 10 μM. (**B**) Wound healing ability of arecoline-treated S-G cells. Top, representative fields; bottom, quantitation. (**C**) SP600125 or PD98059 suppressed arecoline-induced *TNF-α* transcription. (**D**) SP600125 enhanced arecoline-induced *IL-6* transcription but PD98059 did not. Columns, mean of more than triplicate analysis; bars, SE. * *p* < 0.05 and ** *p* < 0.01 indicate the significance levels of arecoline treatment groups compared to the control group. + *p* < 0.05 and ++ *p* < 0.01 indicate the significance levels compared within the arecoline-treated groups.

In our study model, S-G cells was treated with arecoline to represent the early stage of tumorigenesis in the oral cavities of patients who frequently chew areca nuts. While arecoline treatment discouraged cell proliferation and wound healing in S-G cells, it up-regulated the expression of KRT6 and the intracellular levels of ROS and of proinflammatory cytokines. In our experiment, we utilized the MEK inhibitor (PD98059) to suppress arecoline-induced KRT6 expression, with the JNK inhibitor (SP600125) serving as the control group. We observed changes in KRT6 localization and expression level, cell migration, and proinflammatory cytokines. Observing under confocal microscopy, the arecoline-induced KRT6 expression in the SG cells showed no effect and was reduced in the SP600125 and PD98059 pretreatments, respectively (Figure 5A). Suppression of these two signaling pathways did not have a rescuing effect on the decreased cell proliferation or delayed wound healing in S-G cells caused by arecoline treatment. Both SP600125 and PD98059 further inhibited the wound healing ability of arecoline-treated S-G cells, suggesting both JNK and MEK signaling pathways are involved in the intrinsic mechanism of motility in epithelial cells (Figure 5B). Pretreating S-G cells with both SP600125 and PD98059 for 2 h before adding arecoline to S-G cells down-regulated the *TNF-α* gene transcription (Figure 5C). However, pretreating S-G cells with SP600125 but not with PD98059 up-regulated the *IL-6* gene transcription (Figure 5D). Therefore, arecoline treatment increased proinflammatory cytokines in immortalized human gingival epithelial cells, and inhibition of JNK signaling might magnify the *IL-6* gene transcription of arecoline.

### 3.6. The Expression Levels of KRT6 Increased in the Tumor Specimens of Head and Neck Cancer Patients

**Figure 6:**
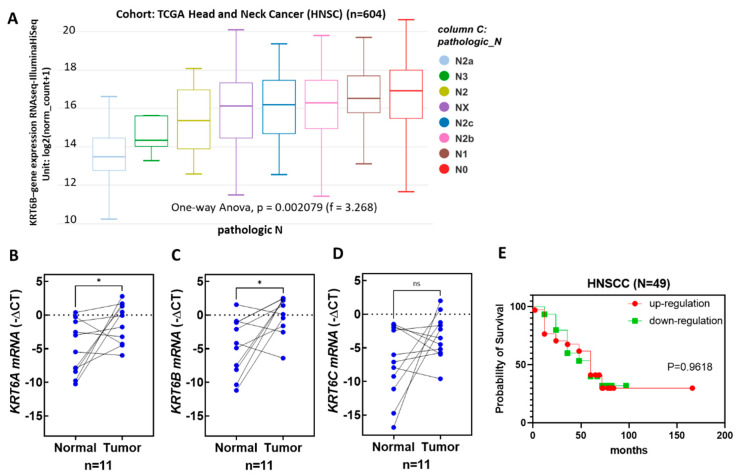
The mRNA expression levels of *KRT6A*, *KRT6B*, and *KRT6C* in paired noncancerous matched tissues and head and neck tumor tissues. The mRNA expression levels of *KRT6A*, *KRT6B*, and *KRT6C* in the paired normal and tumor tissues of oral tumor patients were detected using reverse transcription and quantitative polymerase chain reaction. (**A**) TCGA analysis; *KRT6B* expression associated to pathologic N. to analyze the mRNA expression level of (**B**) *KRT6A*, (**C**) *KRT6B*, and (**D**) *KRT6C* in the same 11 paired normal and head and neck tumor tissues. (**E**) Compared the overall survival curve of low and high *KRT6B* expression groups in 49 paired normal and head and neck tumor tissues. Blue dots, −∆Ct of target genes in normal or tumor tissues; * *p* < 0.05 and ns indicate the significance and no significance of tumor groups compared to the normal group, respectively.

*KRT6* genes mainly include *KRT6A*, *KRT6B*, and *KRT6C*. Analysis of open data from TCGA reveals significant differences in the pathological N for *KRT6B* in head and neck cancer tumors, while *KRT6A* and *KRT6C* show no significant differences (Figure 6A). To verify the results of head and neck cancer in Taiwan, 11 paired normal tissues and tumor specimens of oral cancer patients were collected, and the total RNA was extracted from those tissue samples. The *KRT6A*, *KRT6B*, and *KRT6C* mRNA expression levels were detected by RT-QPCR. Compared to that of the paired normal tissues, the mRNA expression levels of *KRT6A* and *KRT6B*, but not that of *KRT6C*, were significantly increased in the paired tumor tissues (Figure 6B–D). Subsequently, with an increase to 49 pairs of head and neck cancer samples, the analysis of *KRT6B* in relation to patient factors such as smoking, alcohol consumption, areca nut usage, tumor pathology TNM, tumor stage, and survival rate showed no statistically significant differences (Figure 6E).

## 4. Discussion

In our results, arecoline concentrations lower than 12.5 µg/mL did not have a negative effect on cell viability. The protein expression levels of KRT6 in S-G cells were up-regulated after arecoline treatment. This cellular response of KRT6 deposit corresponds to the thickening epithelium layer and tissue keratinization, which are the two main characteristics of the early stage of normal cell transformation and tumor progression [32]. The increased mRNA expression levels of *KRT6A* and *KRT6B* in the paired tumor tissues of our oral cancer patients also confirmed this clinical observation (Figure 6B,C). Pretreating S-G cells with an MEK/ERK inhibitor (PD98059) before adding arecoline decreased KRT6 levels. Therefore, the MEK/ERK signaling pathway, but not that of JNK, is involved in KRT6 synthesis, and PD98059 has the potential to be clinically applied for the prevention of normal cell transformation and oral tissue keratinization [33,34]. The results of this study are corroborated with the literature.

One of the frequent pathogenic causes for many types of diseases is the increased intracellular levels of ROS resulting from exogenous environmental stresses. Chewing areca nut elevates the pH > 8 in the oral cavity, leading to the rapid oxidation of phenolic compounds and resulting in a significant increase in oxygen free radicals and hydrogen peroxide [35]. The free radicals are believed to play a crucial role in the carcinogenic mechanism of areca nut [36]. When S-G cells were treated with arecoline, the intracellular ROS levels were increased. Multiple molecules involved in antioxidative stress, including SOD1, catalase, and GPx4, were also up-regulated (Figure 1C). When the excessive ROS cannot be effectively cleared by anti-ROS enzymes, oxidative damage is inflicted upon molecules within the cell, including lipids, proteins, and DNA [37]. ROS has been demonstrated as a potent inducer of DNA damage and tumor progression [13,38,39]. S-G cells still possess some features of normal epithelial cells, allowing this cell line to produce antioxidative molecules upon one-time exposure to low concentrations of arecoline, leading to the reduction in ROS. Therefore, the turnover capacity of the cellular machinery in oral epithelial cells responsible for producing antioxidative enzymes and transcription factors may be rapidly exhausted upon continuous exposure to various concentrations of areca nuts in the oral cavities. This exhaustion can lead to the transformation of normal epithelial cells and tumor progression.

The cell proliferation ability is an important function of the normal epithelial cells, since the epithelium layers lining the interfaces between organ cavities and the environment periodically need new cells to replace the aging ones or to heal trauma for maintaining normal physiological functions of the organs. A low dose of arecoline treatment has a cell-proliferation-promoting effect on some cancer cell lines [40]. Therefore, it is speculated that prolonged exposure to arecoline may have an impact on cell proliferation. The MEK/ERK pathway is known to signal the proliferation-promoting function, which was activated at 8 h but inhibited at 18 and 24 h after arecoline treatments (Figure 4A). However, arecoline treatment inhibited cell migration but not proliferation of S-G cells (Figure 3), and PD98059 significantly magnified this inhibition of cell migration (Figure 5B). As arecoline demonstrated an inhibitory effect on the ability of migration of S-G cells, this alkaloid can compromise the defending ability of normal epithelial cells. However, S-G cells are derived from gingival epithelium. Further experiments are needed to verify whether the response of buccal mucosa epithelial cells to areca nut is consistent with S-G cells.

IL-6 and TNF-α promote the proliferation and migration of keratinocytes [18,40,41,42], and IL-6 also promotes the reorganization of the keratinocyte cytoskeleton in culture [42]. Since the expression levels of proinflammatory cytokines (IL-6 and TNF-α) were also up-regulated after arecoline treatment (Figure 1D,E), these findings correspond to the hypothesis that inflammation leads to the up-regulation of ROS, favoring the intracellular condition of oxidative damage. Upon activation of TNF-α and IL-6, there is a promotion of epithelial cell movement and proliferation, thereby facilitating wound healing [18,41,42]. However, when *TNF-α* and *IL-6* in epithelial cells are activated by arecoline, it inhibits cell movement but not proliferation (Figure 1 and Figure 3). These findings suggest that low-dose arecoline may influence other factors associated with cell movement and proliferation, such as Wnt signaling [43] and microRNAs [44]. Even at low doses, accumulated arecoline still induces DNA damage in cells and triggers DNA repair processes [40]. DNA damage can also stimulate IL-6 production [45]. Consequently, within the cells, there is a substantial transcriptional activation of the *IL-6* gene to initiate DNA repair processes [46]. The experimental results reveal a significant up-regulation of the arecoline-induced *IL-6* gene transcription in the presence of the JNK inhibitor SP600125 (Figure 5D). It is postulated that this effect is attributed to the immediate activation of IL-6 following DNA damage caused by arecoline. Simultaneous inhibition of JNK prevents the initiation of DNA double-strand break repair, leading to the accumulation of DNA damage [47].

In the in vitro experiments, KRT6 expression increased under areca nut stimulation. *KRT6* genes mainly include *KRT6A*, *KRT6B*, and *KRT6C* [48]. They are both located on chromosome 12, with a 98% similarity in amino acid sequences. When comparing 11 pairs of head and neck cancer tissues with normal tissues, both KRT6A and KRT6B showed a significant increase in tumor tissues. KRT6C expression is also elevated in tumors compared to normal tissues (8/11 T > N), although the differences in KRT6C expression among the groups are substantial and currently lack statistical significance (Figure 6D). This suggests a strong correlation between KRT6 and areca-nut-induced oral cancer. However, an analysis of 49 pairs of head and neck cancer tissues did not reveal associations between KRT6 expression and areca nut consumption, pathological TNM stage, tumor stage, overall survival, etc. (Figure 6E). Possible reasons for this could be a limited sample size, the inclusion of non-oral cancer samples, and incomplete medical records. Additionally, the risk factors for oral cancer patients in Taiwan are not solely areca nut consumption; other factors, such as cigarette smoking and alcohol consumption, are often present [49]. Therefore, future studies should consider increasing the sample size, excluding interfering factors like nasopharyngeal and oropharyngeal cancers, and ensuring more comprehensive medical records for a more accurate analysis.

**Figure 7:**
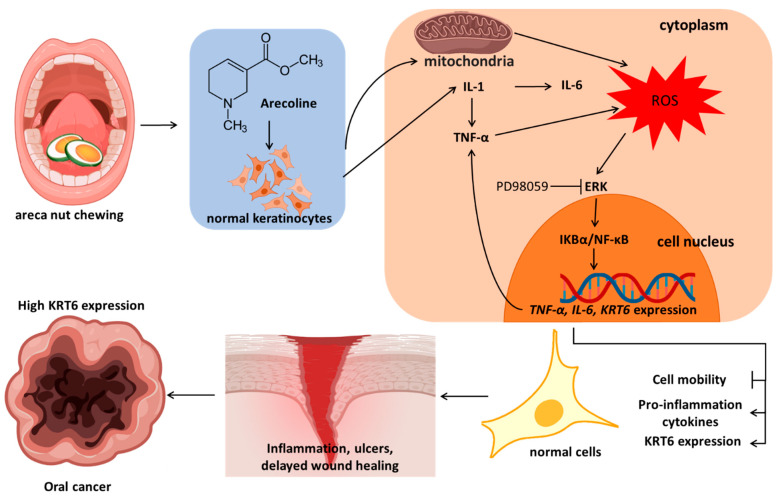
The potential mechanism of arecoline to induce inflammation, ulcers, delayed wound healing, and oral cancer.

The chemical stimulation during areca nut chewing, simulated in vitro by arecoline stimulation of S-G cells, has been verified to induce ROS primarily from mitochondria in human oral mucosal fibroblasts [50]. In vitro experiments have demonstrated that arecoline induces the secretion of interleukin-1 (IL-1) in human oral epithelial cancer cells [51]. The products of keratinocytes activated by IL-1 include TNF-α and IL-6 [52]. Based on previous research reports and the results of this study, the effects of arecoline on normal oral keratinocytes can be inferred as follows (Figure 7): arecoline induces the generation of ROS in cellular mitochondria, leading to the secretion of proinflammatory cytokines such as increased IL-1, thereby inducing the secretion of IL-6 and TNF-α. ROS also activates MAPK/ERK signaling, promoting the nuclear entry of NFκB to enhance the transcription of *KRT6*, *IL-6*, and *TNF-α* genes. The results of this study are consistent with the induction of *KRT6* transcription in human epidermal keratinocytes by IL-1 [53]. The expression of TNF-α establishes a continuous positive feedback loop in the activated signaling. The overall impact of arecoline on cells includes the inhibition of cell migration, promotion of proinflammatory cytokine release, and increased expression of KRT6. These results help explain how areca nut chewing causes recurrent inflammation, delayed wound healing, and ultimately leads to the observed overexpression of KRT6 in oral cancer.

## 5. Conclusions

Chewing areca nut has an impact on oral mucosa, simulated through the stimulation of normal oral epithelial cell line S-G using arecoline. Arecoline stimulates the production of inflammatory cytokine IL-1 and mitochondria, resulting in the generation of ROS. IL-1 promotes the expression of TNF-α and IL-6. ROS activates the MAPK/ERK signaling pathway, leading to the activation of IκB/NFκB, which enters the cell nucleus and initiates the transcription of *KRT6*, *TNF-α*, and *IL-6* genes. TNF-α itself can further enhance ROS production, sustaining the activation process. Taken together, these changes indicate that arecoline induces an inflammatory response, inhibits wound healing, leads to recurrent ulcers, and progresses into oral cancer.

## Data Availability

Data are contained within the article.

## Supplementary Materials

The following supporting information can be downloaded at: https://www.mdpi.com/article/10.3390/biomedicines12020412/s1, Table S1: Information of Primary Antibodies for Western Blot Analysis; Table S2: Primer sequences for RT-qPCR.
