# Supplementary material for: Arecoline Induces ROS Accumulation, Transcription of Proinflammatory Factors, and Expression of KRT6 in Oral Epithelial Cells
Source: Biomedicines. 2024 Feb 9;12(2):412. doi: 10.3390/biomedicines12020412 (PMC10887121; doi:10.3390/biomedicines12020412)
Supplement: Supplementary file 1 [file biomedicines-12-00412-s001.zip › biomedicines-2828574-supplementary.pdf]

Supplementary Materials:

Supplemental Table S1. Information of Primary Antibodies for Western Blot Analysis.

Target	Catalog Number	Supplier
Catalase	sc-271803	Santa Cruz
CDK1	ab265590	abcam
Cyclin B1	#4138	cell signaling
E-cadherin	sc-8426	Santa Cruz
GAPDH	NB300-221	Novusbio
GPx4	#52455	cell signaling
Keratin 6	sc-166074	Santa Cruz
Nrf-2	#12721	cell signaling
p21	#2947	cell signaling
p-ERKthr202/tyr204	#9101	cell signaling
p-IKBαser32/36	GTX32224	GeneTex
p-JNKthr183/tyr185	#4668	cell signaling
SOD1	#37385	cell signaling
Vimentin	sc-6260	Santa Cruz

Supplemental Table S2. Primer sequences for RT-qPCR.

Gene	Sense (5′ –3′)	Antisense (5′ –3′)
TNF-α	*CTCTTCTGCCTGCTGCACTTTG*	*ATGGGCTACAGGCTTGTCACTC*
IL-6	*ACTCACCTCTTCAGAACGAATTG*	*CCATCTTTGGAAGGTTCAGGTTG*
GAPDH	*TGGTATCGTGGAAGGACTCATGAC*	*ATGCCAGTGAGCTTCCCGTTCAGC*
